# A Ringed Fascia Lata Graft Without Peritendinous Areolar Tissue Encircling the Levator Veli Palatini and Superior Pharyngeal Constrictor Muscles Gradually Shrinks to Reduce Velopharyngeal Incompetence, Functioning as an Intravelar Palatal Lift

**Published:** 2013-06-21

**Authors:** Kenya Fujita, Kiyoshi Matsuo, Shunsuke Yuzuriha

**Affiliations:** Department of Plastic and Reconstructive Surgery, Shinshu University School of Medicine, Matsumoto, Japan

## Abstract

**Introduction:** We have previously reported that fascia lata grafts with peritendinous areolar tissue used to treat severe congenital blepharoptosis gradually shrink within 6 weeks postoperatively and maintain long-term shrinkage of 15.5% on average. Accordingly, it seemed possible that a fascia lata graft without peritendinous areolar tissue would shrink more than the one with peritendinous areolar tissue in a clinical setting. We evaluated this possibility in a patient with Klippel-Feil syndrome having postoperative deep atonic nasopharynx. **Methods:** In combination with intravelar veloplasty and palatal lengthening with modified bilateral buccinator sandwich pushback, a ringed fascia lata without peritendinous areolar tissue encircling the levator veli palatini and superior constrictor muscles was grafted to cure severe velopharyngeal incompetence. **Results:** Obstructive sleep apnea did not occur following surgery. Pharyngoscopy, videofluoroscopy, and nasometry showed no amelioration of velopharyngeal incompetence at 1 month postoperatively, but marked velopharyngeal incompetence reduction was evident at 4 months and 2 years after surgery. **Conclusions:** The extended recovery period suggests that the anticipated postoperative shrinkage of the ringed fascia lata without peritendinous areolar tissue played a more prominent role than intravelar veloplasty and palatal lengthening, which posteroinferiorly elongated the atonic soft palate. Although the pharyngeal flap procedure is the most popular technique for treatment of velopharyngeal incompetence, it is sometimes accompanied by respiratory complications. Thus, the gradual postoperative shrinkage of a ringed fascia lata graft encircling the velopharyngeal muscles functions as an intravelar palatal lift and may be an additional surgical method with less respiratory complications to narrow atonic nasopharyngeal port.

We have previously reported that fascia lata grafts with peritendinous areolar tissue used to treat cases of severe congenital blepharoptosis with frontalis suspension gradually shrink within 6 weeks postoperatively and maintain long-term shrinkage of 15.5% on average.^1^ We also reported that anterior tibial tendon grafts with and without peritendinous areolar tissue in rats showed 1-month postoperative shrinkage of 22.9% and 48.6%, respectively, and 5-month postoperative shrinkage of 35.7% and 58.6%, respectively.^2^ Since the vascular network of the peritendinous areolar tissue, which may have contributed to early revascularization of the graft, was removed, tendon grafts without peritendinous areolar tissue appeared to decrease survival of the endotendinous tissue and increase postoperative shrinkage. Accordingly, we aimed to clinically evaluate the possibility that a fascia lata graft without peritendinous areolar tissue would gradually shrink more than the one with peritendinous areolar tissue to narrow postoperative deep atonic nasopharyngeal port in a patient with Klippel-Feil syndrome.

## METHODS

**Figure 1:**
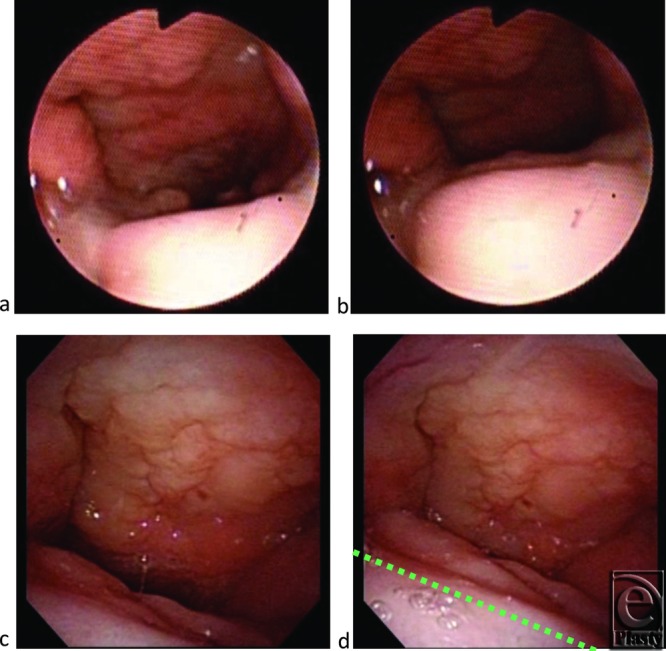
Pharyngoscopic views. Before surgery, resting position (*a*) and on phonating /i/ (*b*). Two years after surgery, resting position (*c*) and on phonating /i/ (*d*). The green dotted line indicates the anterior part of the ringed fascia lata graft.

**Figure 2:**
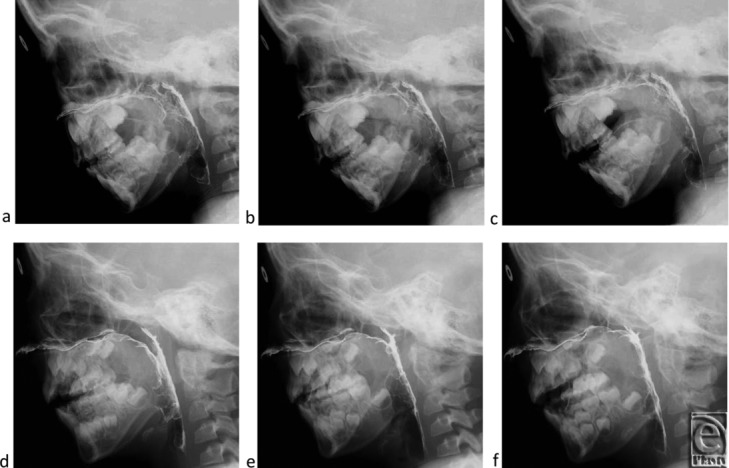
Videofluoroscopic views. Preoperative images at resting position (*a*), on phonating /i/ (*b*), and on phonating /ka/ (*c*). Two-year postoperative images at resting position (*d*), on phonating /i/ (*e*), and on phonating /ka/ (*f*). The diameter of the ring attached to the forehead indicates 10 mm.

The patient was a 6-year-old girl who had a cleft palate associated with Klippel-Feil syndrome but no psychomotor developmental delay. She had undergone cleft palate repair with Furlow's double opposing Z plasty at 22 months of age. However, her postoperative speech showed severe velopharyngeal incompetence (VPI) characterized by hypernasal speech and abnormal articulation. Although speech therapy was commenced at 36 months of age, neither her speech nor velopharyngeal function showed improvement. Pharyngoscopy demonstrated very weak palatal movements while speaking and blowing (Figs 1a and 1b). Her atonic velopharyngeal closure pattern was coronal and left a large gap during phonation that was not compensated for by the lateral pharyngeal walls. Her soft palate showed weak movements while speaking in videofluoroscopic observation. The velopharyngeal distance from the posterior pharyngeal wall to the nasal surface of the soft palate was 12 mm in a resting state, 9 mm on /i/ phonation, and 6 mm on /ka/ phonation (Figs 2a–2c).

**Figure 3:**
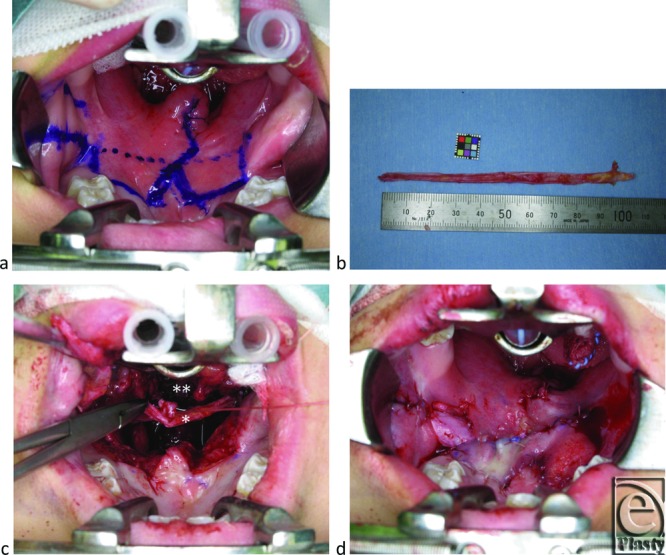
Intraoperative steps. (*a*) The solid and dotted lines indicate the incision line and intended palatal lengthening, respectively, and surround the square mucosal defect. (*b*) A strip of fascia lata. (*c*) The fascia lata graft ends (*) being sutured to each other under the repaired muscles after intravelar veloplasty (**). (*d*) After palatal lengthening, the oral and nasal mucosal defects were covered with the bilateral buccinator flaps.

We first performed palatal muscle re-repair with intravelar veloplasty^3^^-^5 as described by Sommerlad et al,^6^^,^^7^ followed by palatal lengthening with modified bilateral buccinator sandwich pushback as reported by Hill et al^8^ (Fig 3a). Next, a long strip of fascia lata was harvested from her right thigh, using a tendon stripper through a 3-cm skin incision. The harvested fascia lata was 7 mm in width and 11 cm in length, from which the peritendinous areolar tissue was removed to potentially enhance gradual postoperative shrinkage (Fig 3b).^2^ We then prepared a recipient tunnel for the fascia lata graft. Two parallel vertical stab incisions were made in the posterolateral pharyngeal walls to aid passing of the fascial strip. The incisions were carried down to the prevertebral fasicia and were connected by a tunnel under the posterior pharyngeal muscles in front of the atlas. The tunnel was anteriorly extended in front of the levator veli palatini and palatopharyngeus muscles, which were re-repaired. A 2-0 silk suture was then passed through the lateral and posterior tunnel. One end of the graft was tied to the suture, and then the fascia lata was carefully passed through the tunnel (Fig 3c). The ends of the fascia were sutured to each other without tension so as not to leave dead spaces around the levator veli palatini, palatopharyngeus, and superior pharyngeal constrictors muscles. Thus, the ringed fascia lata graft encircled the muscles in front of the atlas. Finally, all surgical wounds were closed with 5-0 vicryl sutures (Fig 3d).

Differences between pre- and postoperative velopharyngeal closure were evaluated by pharyngoscopy, videofluoroscopy, and nasometry, as well as by speech pathologists. Nasometry is a simple diagnostic test using a nasometer (Kay Pentax Elemetrics Nasometer II Model 6400; Lincoln Park, NJ) and provides a “nasalance” score, which is a numeric ratio of nasal to nasal-plus-oral acoustic energy (%).^9^ Vowel and oral test word hypernasality were used to assess reduction of hypernasality. Nasal test word hypernasality was used to assess worsening of hyponasality.

## RESULTS

Since the fascial lata was ringed without tension caused by squeezing, no dead spaces were left following surgery and no accumulation of blood or exudate was observed. Obstructive sleep apnea did not occur despite the presence of postoperative swelling and slight augmentation of the pharynx by the fascia lata graft.

Postoperative pharyngoscopic findings showed narrowing of the pharyngeal port at rest and complete closure of the port during phonation and speech (Figs 1c and 1d).

Videofluoroscopy also showed sufficient elevation of the soft palate at rest, which reduced the amount of palatal movement necessary to completely close the pharyngeal port. Her velopharyngeal distance improved to 7 mm in a resting state and showed complete closure of the pharyngeal port during phonation and speech (Figs 2d-2f).

Regarding perceptual speech analysis by pathologists, the patient's phonation was noted to have greatly improved and her speech had normalized. In particular, her glottal stop articulation disorder had disappeared except for /k/ phonation, which was probably due to mislearning.

Hypernasality had worsened at 1 month postoperatively, likely from swelling, but slowly reduced to a normal range with gradual postoperative shrinkage of the ringed fascia lata graft. Hyponasality did not worsen after surgery. The time-series data of her nasalance scores confirmed improvement (Table 1).

## DISCUSSION

Intravelar veloplasty reinforced velar muscle power, and the palatal lengthening with modified bilateral buccinator sandwich pushback posteroinferiorly elongated the soft palate. However, these procedures appeared not to enhance closure of the velopharyngeal port. The patient's gradual reduction of VPI after an extended period of time suggested that the anticipated postoperative shrinkage of the ringed fascia lata without peritendinous areolar tissue played a more prominent role than the intravelar veloplasty and the palatal lengthening with modified bilateral buccinator sandwich pushback. The shrunken ringed fascia lata appeared to function as an intravelar palatal lift^10^^-^12 and maintained the soft palate in the raised and posteriorly displaced position as well as the lateral and posterior pharyngeal walls in the medially and anteriorly displaced positions. Since nasometry results indicated that hypernasality had reduced but hyponasality did not worsen, the postoperative narrowing of the velopharyngeal port appeared to be optimal.

**Figure 4:**
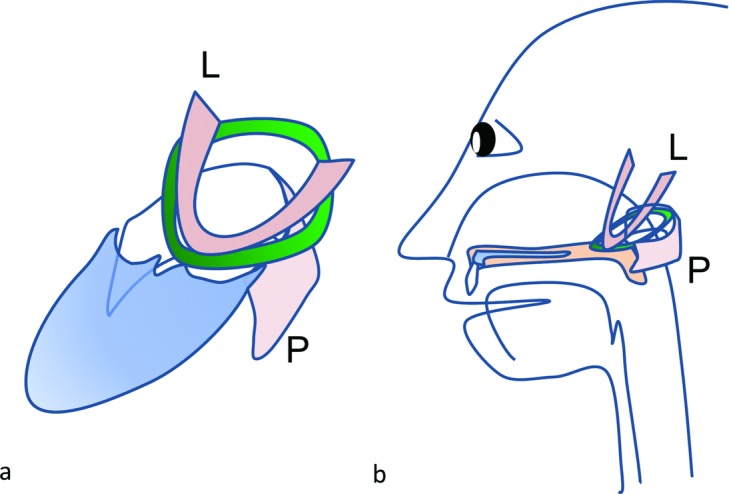
Oblique translucent (*a*) and lateral (*b*) views of the schematic correlation between the muscles and the ringed fascia lata graft (green). L: levator veli palatini muscle; S: superior pharyngeal constrictor muscle.

Although infrequent, VPI following primary cleft palate repair can present a severe problem for patients with deep nasopharynx and powerless velopharyngeal muscles, as seen in this case. The pharyngeal flap procedure is the most popular and reliable technique for postoperative VPI, but it is sometimes accompanied by respiratory complications such as hyponasality, difficulty with nasal respiration, and sleep apnea, all of which may be difficult to secondarily adjust.^13^^-^17 We showed how a ringed fascia lata graft encircling the velopharyngeal muscles may be an additional surgical method to narrow postoperative deep atonic nasopharynx with less risk of respiratory complications (Fig 4).

Klippel-Feil syndrome is a complex disorder of osseous and visceral anomalies that include the classical clinical triad of short neck, limitation of head and neck movements, and low posterior hairline. Cleft palate associated with Klippel-Feil syndrome is reported to be common, and speech results are relatively poor as anomalies of the upper cervical column and cranial base, including the palatal shelves, impede velopharyngeal valving.^18^ Since our patient with Klippel-Feil syndrome had poor palatal and lateral and posterior pharyngeal wall movements, we used a ringed fascia lata sling in the velopharyngeal wall as an intravelar palatal lift instead of a pharyngeal flap. Although her pharyngeal port closure pattern was coronal, there was little compensative movement of the lateral and posterior walls during phonation and even while blowing. Therefore, if we had performed pharyngeal flap surgery, a very broad flap would have been needed to functionally close the pharyngeal port and the procedure would have had a relatively high risk of respiratory complications.

The method of a ringed fascia lata graft for velopharyngeal closure has already been established. Thompson et al and other groups reported a palmaris longus muscle graft for palatal function repair expected reinnervated muscle contraction^19^^-^21 and obtained good speech results with a better functioning palatal reconstruction. The effectiveness of their procedure seemed to depend on the static sling, which narrowed the pharyngeal port by cicatricial contracture of tendon and muscle and functioned in the same way as our ringed fascia lata procedure, using postoperative shrinkage. Gold and Song^22^ also described a palmaris longus tendon transplant without the muscle portion for pharyngopalatoplasty, using a method that greatly influenced our own. Although their 13 patients achieved good results, the authors described the necessity of functional, contractile muscle in the soft palate for this technique to augment the dynamic sphincter mechanism. Indeed, functional muscle contributes significantly to any pharyngoplasty, even the pharyngeal flap. Our fascia lata sling procedure combined with intravelar veloplasty and palatal lengthening with modified bilateral buccinator sandwich pushback could render the atonic velopharyngeal muscles active not by squeezing the velopharyngeal port but by an intravelar palatal lift. There is another report that describes the use of the fascia lata for functional correction of persistent VPI by reconstruction of palatal aponeurosis.^23^ However, this surgical concept is fundamentally different from our own.

The ringed fascia lata graft for an intravelar palatal lift seems to have multiapplicational possibilities for other clinical conditions, such as congenital VPI and submucosal cleft palate with deep pharynx. This graft also has the advantage of being adjustable; if the narrowing of the velopharyngeal port is too tight, we can release or cut the fascia. Furthermore, if VPI reduction is incomplete or the patient needs the additional pharyngeal flap surgery, we can shorten the fascial sling or possibly elevate the superiorly based pharyngeal flap.

## CONCLUSION

A ringed fascia lata graft encircling the pharyngeal musculature combined with intravelar veloplasty and palatal lengthening with modified bilateral buccinator sandwich pushback appears to be a beneficial method to treat VPI due to deep and atonic nasopharynx. Further study is needed on the exact shrinkage rate of fascia lata graft without peritendinous areolar tissue.

## Figures and Tables

**Table 1:** Nasolance score assessment of hypernasality*

	Preoperatively	1 month postoperatively	4 months postoperatively	2 years postoperatively
Vowel hypernasality	43%	61%	27%	29%
Oral test word hypernasality	65%	70%	23%	8%
Nasal test word hypernasality	76%	61%	45%	66%

*Vowels: average scores of /a/, /e/, /i/, /o/, and /u/. Oral test words: average scores of gakko (school), taiko (drum), budo (grape), and jidousha (car). Nasal test words: average scores of “mamimumemo” and “naninuneno” (parts of the Japanese sound table).
